# Effect of systemic antiresorptive medication on the histopathological parameters of implant osseointegration in an in vivo rodent study

**DOI:** 10.1186/s12903-023-02763-z

**Published:** 2023-02-22

**Authors:** Kristian Kniha, Benita Hermanns-Sachweh, Stephan Christian Möhlhenrich, Florian Peters, Marius Heitzer, Philipp Winnand, Frank Hölzle, Ali Modabber

**Affiliations:** 1 Department of Oral and Cranio-Maxillofacial Surgery, University Hospital RWTH Aachen, Pauwelsstraße 30, 52074 Aachen, Germany; 2 Private Clinic for Oral Surgery Dres, Kniha, Rosental 6, 80331 Munich, Germany; 3 Private Institute for Implant Pathology, ZBMT, Campus Melaten, Pauwelsstaße 17, Aachen, Germany; 4 Department of Orthodontics, University of Witten/Herdecke, Alfred-Herrhausen Str. 45, 58455 Witten, Germany

**Keywords:** Dental implant, Zirconia, Titanium, Bisphosphonate, BIC, Osseointegration

## Abstract

**Background:**

The purpose of this study was to evaluate the osseointegration of zirconia and titanium implants in the rat maxilla in specimens under systemic antiresorptive therapy.

**Materials and methods:**

After 4 weeks of systematic medication administration (either zoledronic acid or alendronic acid), 54 rats received one zirconia and one titanium implants that were immediately inserted in the rat maxilla after tooth extraction. Twelve weeks after implant placement, histopathological samples were evaluated for implant osteointegration parameters.

**Results:**

The bone-implant-contact (BIC) ratio revealed no significant inter-group or inter-material differences. The distance between the implant shoulder to the bone level was significantly greater around the titanium implants of the zoledronic acid group compared to the zirconia implants of the control group (*p* = 0.0005). On average, signs of new bone formation could be detected in all groups, although often without statistical differences. Signs of bone necrosis were only detected around the zirconia implants of the control group (*p* < 0.05).

**Conclusions:**

At the 3-month follow-up, no implant material was demonstrably better than the others in terms of osseointegration metrics under systemic antiresorptive therapy. Further studies are necessary to determine whether there are differences in the osseointegration behavior of the different materials.

## Introduction

Antiresorptive medications, such as bisphosphonates and monoclonal antibodies, interfere with physiological bone metabolism and are intended to inhibit pathological bone resorption [1]. However, bisphosphonates also prevent the renewal of injured bone and have a half-life of approximately 11 years in bone because of their irreversible binding to bone [2]. In 2008, 200 million doses of bisphosphonates were prescribed in Germany [3]. Since the incidence of bisphosphonates increases with the aging of society, these numbers are likely to increase in the coming years [3]. Furthermore, bisphosphonate accumulates in bone over 100 times faster than when administered orally and leads to a higher bone mineralization [4]. The incidence of tumor patients on systemic antiresorptive medication (such as in cases of multiple myeloma, prostate carcinoma, bronchial carcinoma, and breast carcinoma) in Germany alone is around 200,000 new cases per year and rising [5].

If these drugs are used, surgical interventions such as implantation represent a risk for jaw necrosis [6, 7]. Dental implants can be used, but they come with a greatly increased risk of complications due to oral antiresorptive therapy [8]. Patients with low oral doses will have an increased risk of jaw necrosis during implantation; for patients with high doses, for example in the case of systematic medication, no dental implantation is recommended at all according to a recent systematic review [3]. Extensive operations, such as jaw bone augmentations, can represent a contraindication for the risk group mentioned. According to this guideline, patients with a high-risk profile should not be treated with implants, which is why no sufficiently fixed restoration is possible in these cases, and patients have to live with the risk of prosthetic pressure points.

Osseointegration is a special form of bony healing in which vital bone cells and bioserological components bond with the implant surface [9]. Provided that no relevant disturbing influences occur, this connection is permanent. Titanium proved to be a biologically suitable material on which chemical bonds can form with the surrounding tissues, which are also sufficiently stable biomechanically [10]. Among other things, it has been proven based on the first osseointegrated implants can remain in function for over 40 years.

In dental implantology, titanium is considered the gold standard according to numerous long-term studies. The emergence of the all-ceramic, high-performance material zirconium dioxide has raised hopes for a suitable all-ceramic material. Initial preclinical and clinical studies look promising. According to the findings, zirconia ceramic is biocompatible, and the ceramic implant surface is well accepted by the bone tissue. Furthermore, authors concluded that one-piece zirconia implants can be used as an alternative for titanium implants [11–14]. Both titanium and zirconia implant materials showed good osseointegration behavior in a preclinical study and also presented high success and survival rates under physiological condition in patients, even with immeditate implant treatment [15–18]. This inevitably leads to the question of what implant material may have a lower risk of complication under systemic antiresorptive therapy due to the individual material properties. The primary aim of this study was to evaluate the histopathological osseointegration using the bone-implant-contact (BIC) ratio by comparing immediate zirconia and titanium implants in the rat maxilla. Furthermore, peri-implant bone levels and bone behaviors were investigated.

## Material and methods

### Experimental protocol

This animal study enrolled 54 adult male Sprague–Dawley rats, each weighing 250 g and aged 7 weeks (Janvier Labs, Le Genest-Saint-Isle, France). Two experimental groups and one control group, each with 18 animals, were randomly assigned to the following treatments: zoledronic acid (Group 1), alendronic acid (Group 2), and no medicine (Group 3). Systemic antiresorptive medicine was started 4 weeks prior to implantation and continued for 4 months. Before administration, the medicines were diluted in physiologic phosphate-buffered saline. Once a week, rats in Group 1 were given 0.04 mg/kg of body weight of zoledronic acid (Mylan dura GmbH, Darmstadt, Germany) intravenously in the tail vein [19]. The rats in Group 2 were given 0.2 mg/kg of body weight of alendronic acid (alendronate sodium trihydrate, Sigma Aldrich GmbH, Munich, Germany) subcutaneously five times a week [20].

One examiner performed the surgery, and another examiner assessed the histopathological samples using blinded data evaluation. This study was carried out in accordance with the guidelines of the European Parliament and of the Council on the protection of animals used for scientific purposes, Animal Research: Reporting of In Vivo Experiments (ARRIVE) and Directive 2010/63/EU. The study protocol received ethical approved from the appropriate local authority (Landesamt für Natur und Verbraucherschutz, Recklinghausen, Germany; Ref. 2018A314). The rats were given free access to food and water, with only soft moistened food provided following implantation until the end of the study.

### Implant placement

**Fig. 1:**
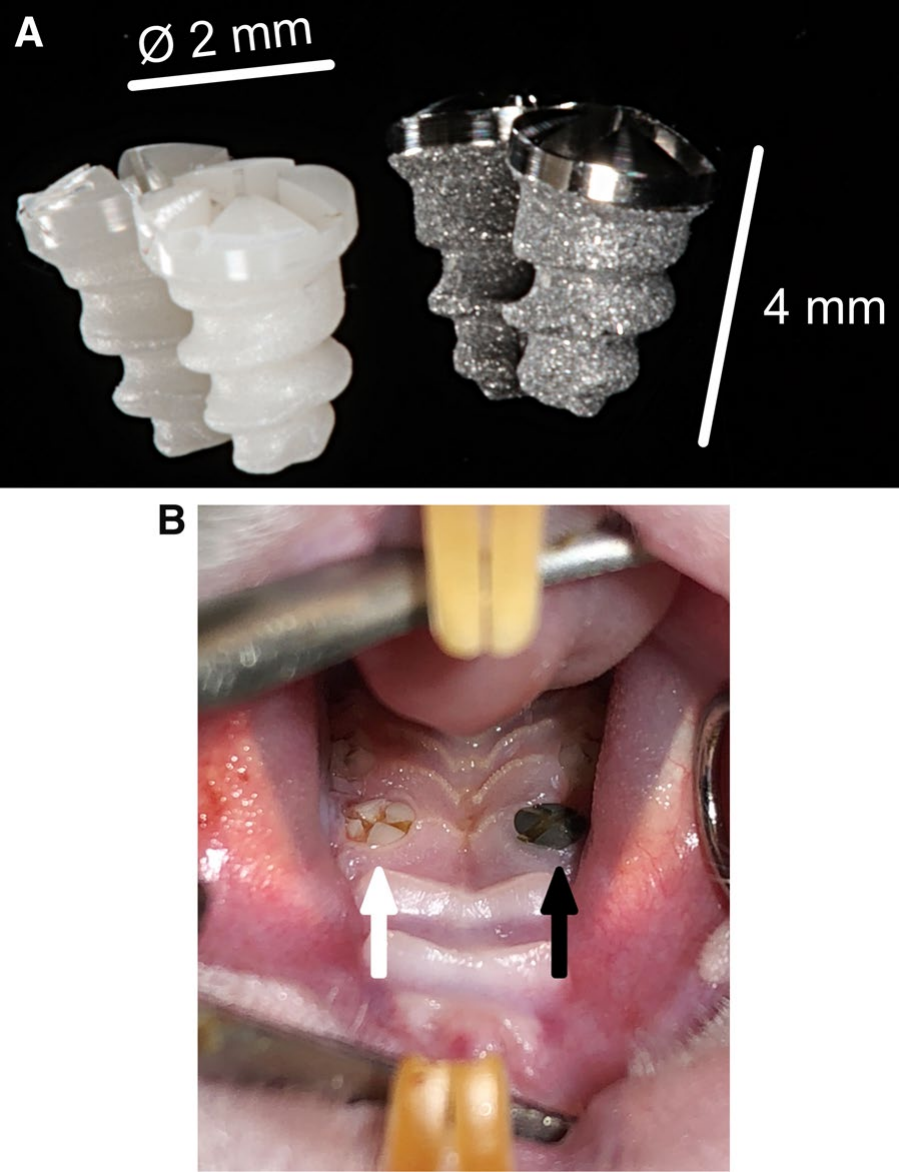
**A** The zirconia and titanium implants had a diameter of 2 mm and a length of 4 mm. **B** Using a split-mouth design, after extraction of the primary molar of the upper jaw on each side, one zirconia and one titanium implants were immediately inserted

Surgery was conducted after 4 weeks of medication administration to both test groups. The Straumann Company (Institute Straumann AG, Basel, Switzerland) custom made a total of 54 microrough titanium or zirconia implants with a polished shoulder (4-mm length and 2-mm diameter, Fig. 1) using the same method as commercially available implants. This surface was generated with a macro roughness by rough sandblasting. Subsequently, a micro-roughness is generated by acid etching. The titanium surface was optimized by sandblasting and the zirconia surface by zirconia particle blasting. Mean surface roughness values of titanium were 1.23 versus zirconia 0.59 (micrometer).

The rodents were injected with an intraperitoneal sedative mixed injection comprised of 90 mg/kg of body weight of ketamine (Medistar GmbH, Ascheberg, Germany) and 0.2 mg/kg of body weight of medetomidine hydrochloride (Domitor, Bayer Austria, Vienna, Austria). A split-mouth design was used. After extraction of the primary molar of the upper jaw on each side, one zirconia and one titanium implants were immediately inserted. The individual implant material was distributed on the maxillary side by randomization. Implantation was performed according to the manufacturer’s guidelines with a transgingival healing procedure. Toward the end of surgery, 0.8 mg/kg of body weight of the antitoxin atipamezole hydrochloride (Orion Pharma, Espoo, Finland) was injected subcutaneously. During the 3 initial postoperative days, the animals received 4 mg/kg of carprofen (Rimadyl, Zoetis GmbH, Berlin, Germany) subcutaneously once per day as indicated by a score sheet. The animals were sacrificed at the end of the study under deep isoflurane anesthesia by cervical dislocation. We then studied the histopathological results of all implants that were incorporated.

### Histopathological sample evaluation

At 12 weeks after implant placement, the animals were killed by cervical dislocation under deep isoflurane anesthesia (4%). The tibia samples were stored in 4% formalin (neutrally buffered with methanol; Otto Fischar GmbH & Co. KG, Saarbrücken, Germany) for 48 h. The samples were dehydrated using an ascending ethanol gradient (50–100%) prior to embedding in methyl methacrylate resin (Technovit 9100, Heraeus Kulzer GmbH, Frankfurt, Germany). Coronal sections of the embedded undecalcified specimens were obtained at a thickness of about 200 µm using the EXAKT cutting unit (EXAKT Technologies Inc., Oklahoma City, Oklahoma, USA), and then they were thinned and polished manually to a final thickness of about 50–70 µm [21]. Final specimens were stained with toluidine blue according to manufacturer’s protocol and were analyzed using digital microscopy. One slide was obtained for each implant in the coronal section through the implant center.

The tissue structures were analyzed by digital microscopy by one specialized pathologist. The set of parameters evaluated in the histomorphometric analysis included a quantitative evaluation of the peri-implant bone. The bone-to-implant ratio (μm) was calculated by measuring the complete circumference of the implant and then recording the area that had histologic bone contact. Using this, the implant contact through bone in relation to the entire surface (BIC ratio) was determined. The distance between the implant shoulder and the peri-implant bone level (mm) was measured on both sides. Additionally, semiquantitative analyses were performed for new bone formation, bone resorption, necrosis, signs of inflammation, and connective tissue proliferation based on a previously published score (0 = normal, 1 = minimal count, 2 = progressing count, and 3 severe amount) [22]. All parameters were examined under 40–600 times magnification with the OLYMPUS digital microscope DSX-1000 (Olympus, Hamburg, Germany) and the integrated morphometric stream desktop software [22].

### Statistical analyses

Analyses were performed using the Prism 8 software (GraphPad, La Jolla, CA, USA) running on Apple OS X. Variables were analyzed using the Kolmogorov–Smirnov normality test. Kruskal–Wallis and Dunn’s multiple comparison tests with adjustment were used to identify differences between parameters. A p value of less than 0.05 was considered statistically significant.

Post-hoc power analysis was performed with the G-Power software (Heinrich-Heine-Universität, Düsseldorf, Germany) using the post hoc ANOVA test by means of groups to determine the power of 100% (primary study aim: BIC) based on the total sample size of 52 with an effect size of 8.7 and an alpha of 0.05.

## Results

**Fig. 2:**
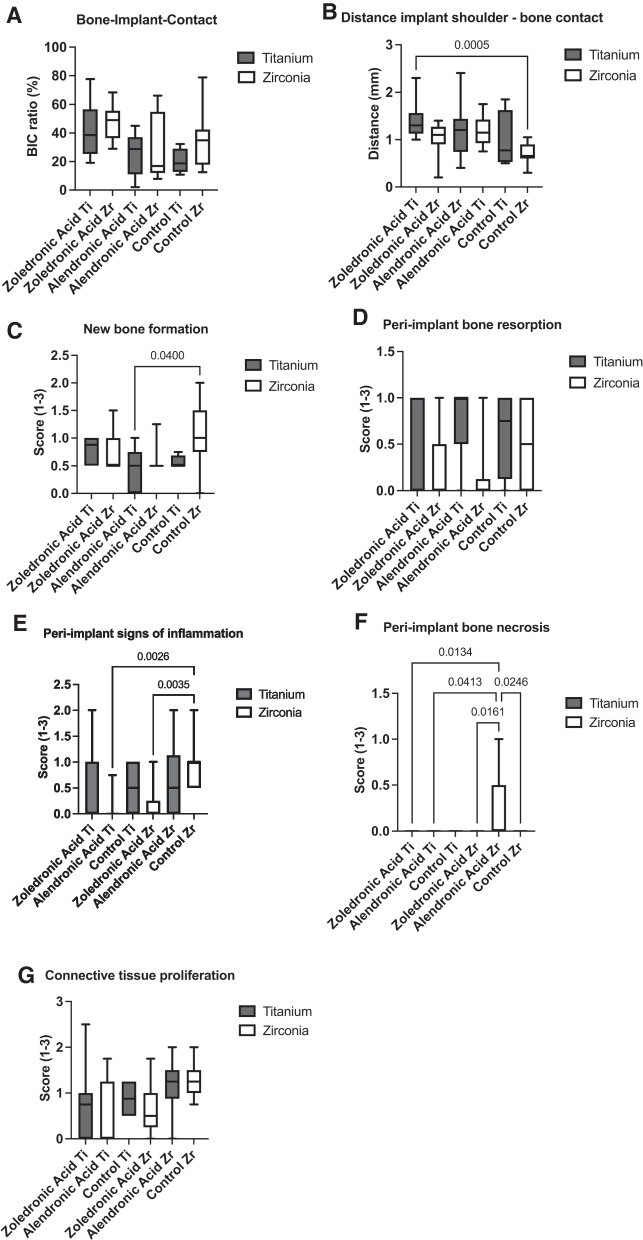
**A** The implant contact through bone in relation to the entire surface of the implant (BIC ratio; %) was determined. **B** The distance between the implant shoulder and the peri-implant bone level (mm) was measured on both sides of the implant. **C** New bone formation, **D** bone resorption, **E** bone necrosis, **F** signs of inflammation, and **G** connective tissue proliferation were evaluated according to a previously published score (0 = normal, 1 = minimal count, 2 = progressing count, and 3 severe amount)

This study investigated histological results in an animal model at one time point at month follow-up. At the end of the test series, the following implants were osseointegrated and used for histopathological evaluation: 15 titanium and 13 zirconia implants in Group 1 (zoledronic acid), 9 titanium and 10 zirconia implants in Group 2 (alendronic acid), and 4 titanium and 11 zirconia implants in Group 3 (control group). At 3 months after implant insertion, the BIC ratio revealed no significant inter-group or inter-material differences (Fig. 2A; *p* > 0.05). Group 1 showed the highest BIC mean values for both materials. However, the titanium implants of Group 2 and zirconia implants of Group 3 showed the lowest BIC ratios (Table 1).

**Table 1:** Descriptive data of the different methods used for the test and control groups (Ti = titanium and Zr = zirconia)

	Zoledronic acid Ti	Alendronic acid Ti	Control Ti	Zoledronic acid Zr	Alendronic acid Zr	Control Zr
Number of values	15	9	4	13	10	11
*BIC ratio%*						
Minimum	19.14	2.00	10.80	28.97	788	12.50
Maximum	77.66	45.00	32.30	68.48	66.21	78.75
Mean	42.28	25.54	20.15	47.49	29.93	34.36
SD	18.47	14.77	8.93	11.38	23.30	19.65
*Distance implant shoulder—bone mm*						
Minimum	1.00	0.75	0.50	0.20	0.40	0.30
Maximum	2.30	1.75	185	1.40	2.40	1.05
Mean	1.38	1.19	0.97	1.00	1.18	0.70
SD	0.37	0.32	0.61	0.35	0.59	0.22
*New bone formation score*						
Minimum	0.50	0.00	0.50	0.50	0.50	0.00
Maximum	1.00	1.00	0.75	1.50	1.25	2.00
Mean	0.76	0.44	0.56	0.75	0.57	1.02
SD	0.24	0.39	0.12	0.32	0.23	0.56
*Bone resorption score*						
Minimum	0.00	0.00	0.00	0.00	0.00	0.00
Maximum	1.00	1.00	1.00	1.00	1.00	1.00
Mean	0.28	0.77	0.62	0.23	0.15	0.59
SD	0.46	0.44	0.47	0.38	0.33	0.43
*Bone necrosis score*						
Minimum	0.00	0.00	0.00	0.00	0.00	0.00
Maximum	0.00	0.00	0.00	0.00	1.00	0.00
Mean	0.00	0.00	0.00	0.00	0.20	0.00
SD	0.00	0.00	0.00	0.00	0.34	0.00
*Signs of inflammation score*						
Minimum	0.00	0.00	0.00	0.00	0.00	0.50
Maximum	2.00	0.75	1.00	1.00	2.00	2.00
Mean	0.39	0.08	0.50	0.15	0.60	0.90
SD	0.62	0.25	0.57	0.31	0.69	0.43
*Connective tissue proliferation score*						
Minimum	0.00	0.00	0.50	0.00	0.00	0.75
Maximum	2.50	1.75	1.25	1.75	2.00	2.00
Mean	0.75	0.55	0.87	0.65	1.15	1.29
SD	0.71	0.71	0.43	0.50	0.56	0.44

The distance of the bone to the implant shoulder presented a significantly value around titanium implants of the zoledronic acid group compared to zirconia implants of the control group (*p* = 0.0005). The remaining group and material comparisons showed no statistical differences (Fig. 2B). A pronounced bone formation according to score was recorded around zirconia implants of the control group compared to titanium implants of Group 2 (Fig. 2C; *p* = 0.04). On average, signs of new bone formation could be detected in all groups, although often without statistical differences.

In addition to the new bone formation, bone resorption around the different implants was also present but did not show any significant differences between groups (Fig. 2D). Signs of bone necrosis around the zirconia implants were detected in the control group (Fig. 2E). The score was significantly higher than in the titanium of Group 1 (*p* = 0.0134), titanium of Group 2 (*p* = 0.0413), zirconia of Group 2 (*p* = 0.0161), and zirconia of Group 3 (*p* = 0.0246).

**Fig. 3:**
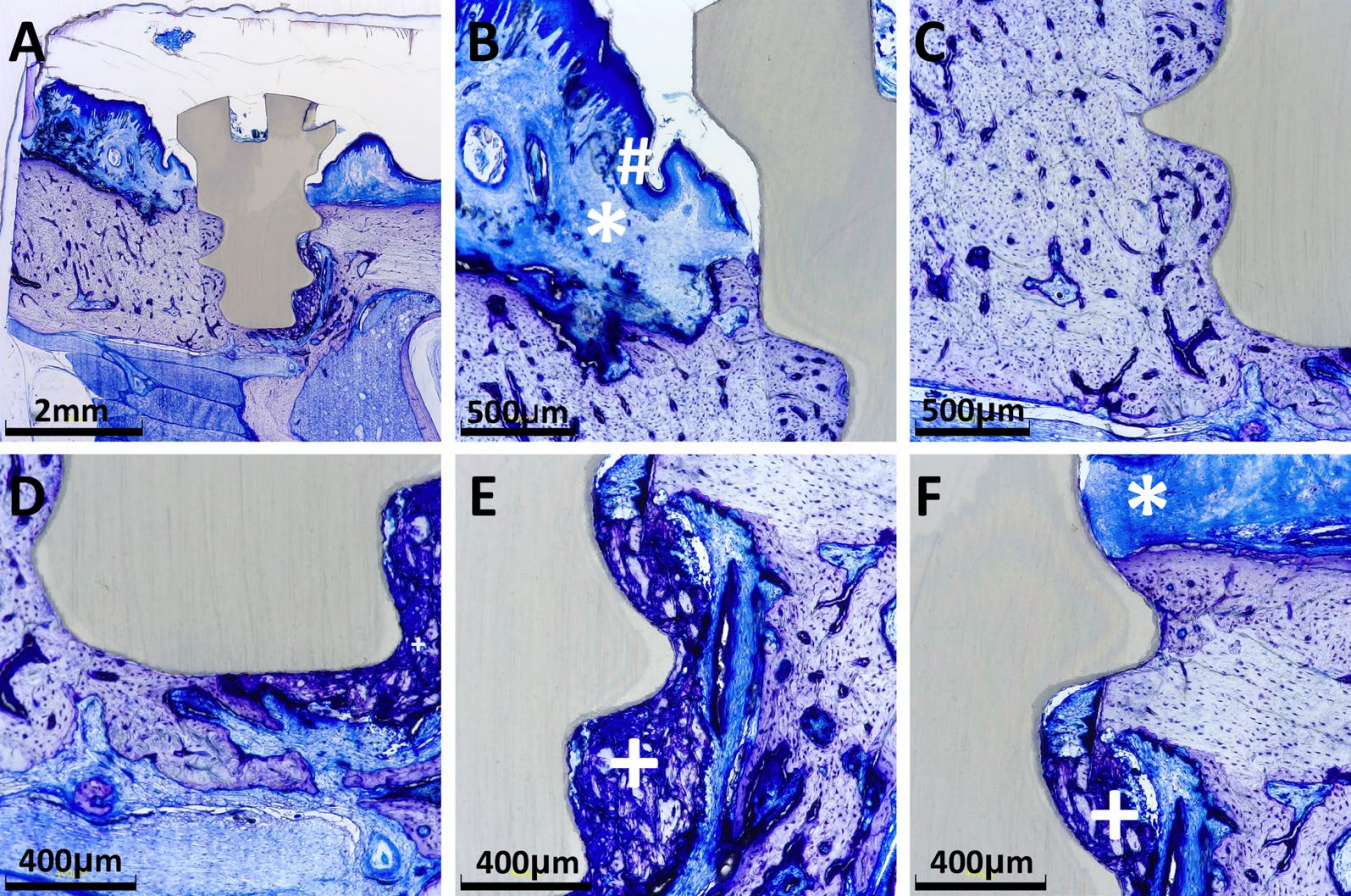
**A** A zirconia implant in the zoledronic acid test group. **B** Squamous epithelium of the gingiva is clearly visible (#). **C**, **D** Implant tip revealed a large BIC area. The adjacent osteoid and new bone formation was detected (+). **E** Focally, new bone formation was present (+). No evidence of inflammatory reaction or necrosis was found. **F** Beside the neck of the implant, circumscribed connective tissue formation was visible (*)

**Fig. 4:**
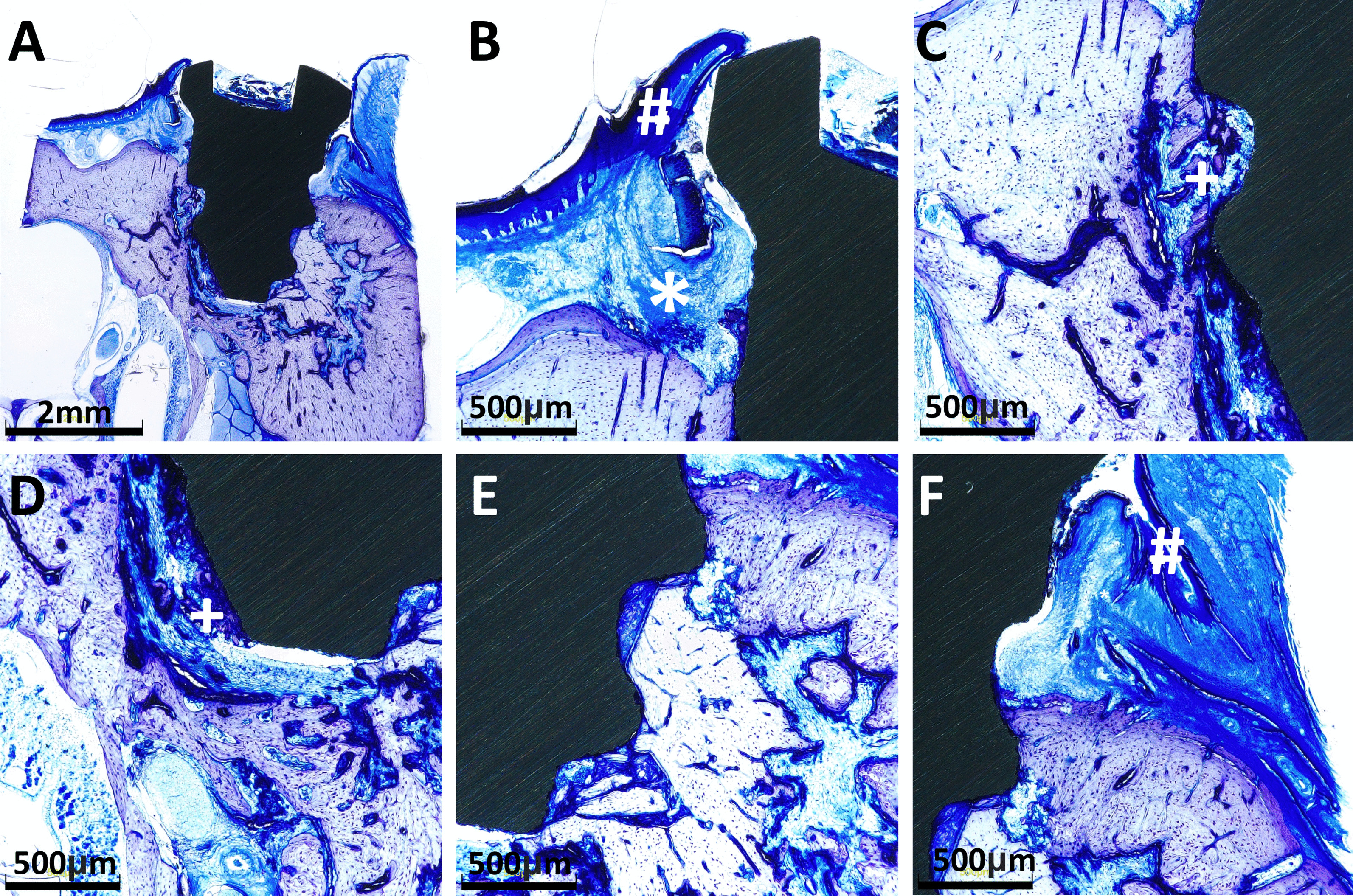
**A** Titanium implant in the zoledronic acid test group. **B** Squamous epithelium of the gingiva (#) and connective tissue (*) adjacent to the implant neck. **C**, **D** Osteoid and bone formation (+) with focal BIC can be seen laterally and in the area of the implant tip. **E** No evidence of inflammatory response or necrosis. **F** Squamous epithelium of the gingiva (#) and connective tissue adjacent to the implant neck

Peri-implant signs of infection were significantly higher around the zirconia implants of the control group compared to the zirconia implants of Group 1 (*p* = 0.0026) and Group 2 (*p* = 0.0035, Fig. 2F). The histomorphometrical analysis revealed no differences regarding connective tissue proliferation (Fig. 2G). Figures 3 and 4 show histological preparations of the zirconia and titanium implants of one rat of the zoledronic acid group. Not only the control groups but also the test groups showed a large BIC area. Squamous epithelium of the gingiva in the area of the implant shoulder and the adjacent osteoid and new bone formation were detected in these examples. No evidence of inflammatory reaction or necrosis was noted in this example of the test group.

## Discussion

This study aimed to evaluate histopathological implant osseointegration comparing immediate zirconia versus titanium implants in the rat maxilla under systemic antiresorptive therapy. An animal model using rats for the study of dental surgical interventions in relation to wound healing and Bisphosphonate-Related Osteonecrosis of the Jaw (BRONJ) was previously described in the literature [23]. Additionally, the rat maxilla’s mesial root socket of the first molar is a good area for dental implant research [24]. At the end of our study, 62 implants were found to be integrated.

Studies have shown that surgical placement of dental implants in humans, regardless of the onset timing of bisphosphonates, is a risk factor for the development of osteonecrosis [25–27]. According to a literature review, the use of antiresorptive drugs increases the risk of osteonecrosis in patients with implants that are subjected to functional loading [28]. In our study, small signs of bone necrosis were detected only around zirconia implants in rats treated with alendronate. Signs of peri-implant infection could be detected in most of the groups, but the differences were not significant. A possible explanation for the low occurrence of necrosis zones is due to the fact that only implants that were still integrated after three months were examined. In the case of BRONJ, the implant may have been lost beforehand.

It has been shown that differences in implant design features influenced the osseointegration pathway [29]. Interactions between proteins, cells and tissues, but also implant surfaces can affect the implant integration. Surface treatments can differ outcomes and the surfaces applied in this study are not the same between zirconia and titanium implants [30]. However, it could be shown in a preclinical study, that both materials osseointegrated equally, so without significant difference [31]. Hou et al. [32] evaluated the osseointegration of titanium implants in rats with systemic zoledronate administration and found that bone-to-implant contact was negatively influenced by the drugs. Similarly, Dikicier et al. [33] demonstrated that there is an unfavorable implant osseointegration regarding the BIC value after administering systemic zoledronic acid in rats. In contrast, our results regarding the BIC ratio showed that there were no significant differences between the test groups and the control group.

Viera-Negron et al. [34] concluded that the osseointegration of implants in the rat maxilla was improved by the systemic administration of alendronic acid. In another study, systemic bisphosphonate delivery enhanced implant osseointegration in 15 animals with induced osteoporotic conditions [6]. Furthermore, in animal models, systemic injection of zoledronate improved osseointegration of orthopedic implants [20, 35]. However, because of the inherent variations between preclinical and clinical populations, the results regarding a meta-analysis should be regarded with caution [36]. The potential risk of bisphosphonate-related BRONJ in patients undergoing dental implant therapy cannot be disregarded.

The final implant survival of this study could be due to various causes. In this animal experiment, occlusal overload could only be excluded to a limited extent, despite soft food. Furthermore, the hygiene of the implants was only conditionally given, since food residues from the soft food could have accumulated around the implants. In addition, the tooth extraction and simultaneous immediate implantation could have led to an overstressing of the bone bed. Nevertheless, according to the inclusion criteria, only integrated implants at the end of the study were enrolled in this assessment.

The aim of this investigation was to analyze a high-risk group for dental implantation due to a systemic antiresorptive medication in the rat model using a high application rate. We applied antiresorptive medication per body weight comparable with that of humans. We recognize that pathophysiology can vary between rodents and humans [37–43]. Intravenous administration, as applied in this study, is associated with a higher risk than oral administration (i.v.—> oral application) [44]. While no single animal model can entirely replicate human bone and joint formation and repair, different species can help with implant development [45]. It should be noted that there are differences in bone behavior between rats and humans. Bone growth in rats lasts significantly longer than it does in humans following sexual maturity [45]. At places like the jaw bones, rats have better bone repair potential than humans. Further limitations were that only histological results with the the “BIC” osseointegration value at one time point were measured. It also should be noted that each specific implant surface individually influences the osseointegration behavior.

## Conclusions

To find out if the various materials behave differently during osseointegration, more preclinical and clinical research is required. Regarding osseointegration parameters under systemic antiresorptive therapy, no implant material was clearly superior to the others at the 3-month follow-up.

## Data Availability

The datasets used and/or analysed during the current study are available from the corresponding author on reasonable request.
